# A global bibliometric and visualized analysis of the status and trends of gastroparesis research

**DOI:** 10.1186/s40001-023-01537-1

**Published:** 2023-11-28

**Authors:** Meng Li, Ning Gao, Shaoli Wang, Yufeng Guo, Zhen Liu

**Affiliations:** 1 Department of Gastroenterology, Guang’anmen Hospital, China Academy of Chinese Medical Sciences, No. 5 Beixiange St., Xicheng District, Beijing, 100053 China; 2 Department of Acupuncture and Moxibustion, Guang’anmen Hospital, China Academy of Chinese Medical Sciences, No. 5 Beixiange St., Xicheng District, Beijing, 100053 China

**Keywords:** Gastroparesis, Bibliometric, Research hotspots, VOSviewer, Citespace

## Abstract

**Background:**

Gastroparesis has a substantial impact on the quality of life but has limited treatment options, which makes it a public health concern. No bibliometric studies on gastroparesis have been published thus far. Thus, this article aims to summarize and analyze research hotspots to provide a reference for clinical researchers.

**Materials and methods:**

Gastroparesis-related research articles were searched in the Web of Science Core Collection (WOSCC), and relevant information was extracted after screening. A total of 1033 documents were analyzed with the bibliometric method using Microsoft Excel, Citespace, and VOSviewer.

**Results:**

Overall, our search retrieved 1033 papers contributed by 966 research institutions from 53 countries. Since 1980, publications in this field have increased rapidly. United States (*n* = 645) and Temple University (*n* = 122) were the most productive country and institution, respectively. Parkman, with 96 publications, was the most prominent author.

**Conclusions:**

Research hotspots in gastroparesis can be summarized into four domains: innovation in diagnostic modalities, change of oral therapeutic agents, choice of surgical interventions, and pathological mechanisms. Future research on gastroparesis should focus on the quality of life of patients, diagnostic techniques, pyloromyotomy, and transpyloric stent placement.

## Introduction

Gastroparesis is a syndrome characterized by delayed gastric emptying (DGE) in the absence of mechanical gastric obstruction [1]. Common symptoms include nausea, vomiting, postprandial fullness, and abdominal distention [2]. About 72% of patients with gastroparesis present with abdominal pain, which is easily overlooked in clinical practice. However, some patients have more insidious symptoms or no discomfort [3]. Epidemiological surveys have shown that the prevalence rate is 13.8 per 100,000 people in the UK [4] and 24.2 per 100,000 people in the US [5]. Nevertheless, in clinical practice, the accurate prevalence of gastroparesis is difficult to determine: only one in nine patients with high-probability gastroparesis receives an accurate diagnosis [6]. Both gastroparesis and functional dyspepsia are gastric neuromuscular disorders, and because of the similarity of their symptoms, they are often confused with each other [7]. The current understanding of the etiology of gastroparesis recognizes three main types of gastroparesis: idiopathic gastroparesis (IG), diabetic gastroparesis (DG), and postoperative gastroparesis (PG) [8]. IG is the most common type of gastroparesis, predominantly affecting female patients [9]. Patients with DG account for about one-third of all cases of gastroparesis [10]. Hyperglycemia has been reported to increase the risk of gastroparesis, which occurs within 10 years of diagnosis in about 5.2% of patients with type 1 diabetes and a lower percentage of those with type 2 diabetes [11]. In addition, there is a link between gastroparesis and Parkinson’s disease [10]. Although the exact pathogenesis of gastroparesis has not yet been fully understood, it is thought to involve the loss of vagal nerve and interstitial cell function [12]. Gastroparesis reduces patients' quality of life and places a significant financial burden on the healthcare system [13].

Bibliometrics is a quantitative method of analyzing the characteristics and trends of research in a given field using previously published academic literature; it was first introduced by Pritchard in 1969 [14]. This method has been widely employed in the fields of information science, chemistry, and physics and has new potential in medicine [15]. No such research that reviews and analyzes the existing research results in the field of gastroparesis has been published so far. An assessment of the current state of research in the gastroparesis field is necessary.

Based on the above information, this study aims to solve the following questions:

1. What are the annual trends of publications in the field of gastroparesis?
2. Which countries, authors, and institutions focus on and contribute the most to the field of gastroparesis?
3. Which journals are more willing to publish articles on gastroparesis?
4. What are the hot research topics in the field of gastroparesis and where are the future research prospects likely to emerge?

Through this study, we hope to help provide essential learning resources for clinicians and investigators less familiar with the field, as well as gain insight into new perspectives and foundations for the future in gastroparesis research.

## Methods

### Data sources and search strategies

**Fig. 1:**
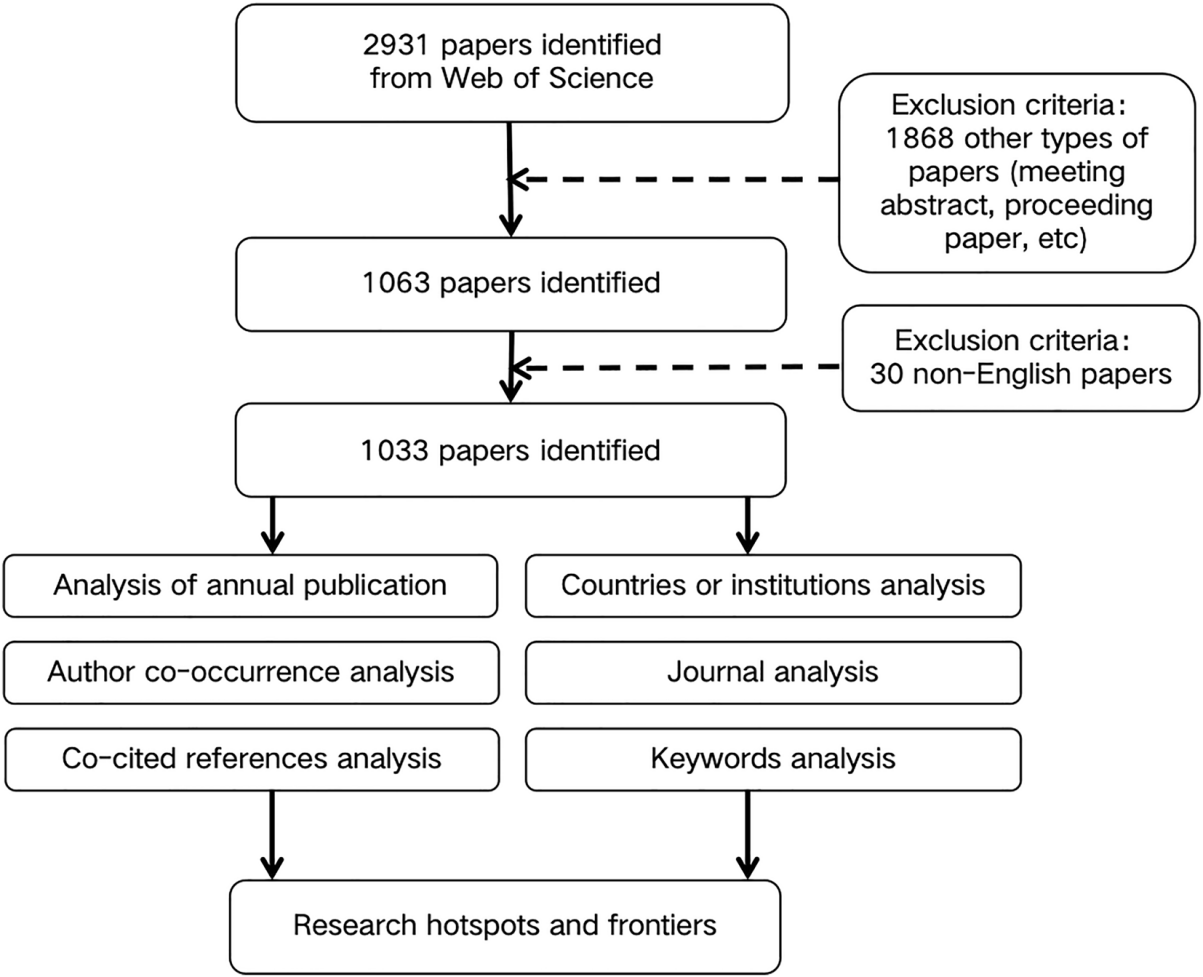
Flow chart of literature screening

Web of Science (WOS) is the earliest, most comprehensive, and most detailed database in the world [16], and it has had a significant impact in the biomedical field [17]. We conducted a search for all publications in the gastroparesis field in the Science Citation Index-Expanded (SCIE) database in the WOS Core Collection (WOSCC), for the period spanning from database inception to November 16, 2022. The search terms were formulated based on the clinical experience, medical subject headings (MeSH), and published articles [18, 19], and a title search was finally performed on the retrieved articles to avoid the inclusion of a large number of unrelated articles [20–22]. The search formula was finally set as follows: TI = (Gastropares*) OR TI = (Gastric Stas*). The language of the article was limited to English, and the type of article was set to either research or review. Two researchers (ML and NG) independently performed the data search, and any differences in opinions were resolved by discussion. On screening the retrieved articles with the selection criteria, 1033 publications in the field of gastroparesis were finally included in the analysis (Fig. 1).

### Data collection

Data regarding the following parameters were downloaded from WOSCC for the publications identified: title, author, journal, institution, publication year, and keywords. The data were exported in plain text format. Impact factor (IF) and journal citation report (JCR) category were obtained through JCR Science Edition (2021) [17]. To avoid bias caused by database updates, data retrieval and export were chosen to be completed on the same day (November 16, 2022).

### Statistical analysis

Analyses of descriptive statistics for countries, institutions, authors, journals, citations, and keywords were performed using Microsoft Excel (Version 2019; Microsoft Corporation; Washington, United States) [23] to produce world heat maps, line graphs, and bar charts. In addition, visual graphs of collaboration networks and keyword clusters were constructed using the VOSviewer (Version 1.6.18; Leiden University; Leiden, Netherlands), which was developed by Van Eck and Waltman at Leiden University in the Netherlands. In the cooperation network, the number of publications determines the size of the node, the connection between nodes indicates the cooperation relationship, and the thickness of the connection reflects the strength of the cooperation [24]. Keyword burst analysis was performed using CiteSpace (Version V; Drexel University; Pennsylvania, United States), to determine the research hotspots and frontier trends from the time dimension [25]. The software parameters were set as follows: time slice (1990–2022), years per slice (1), and selection strategy (g-index, *k* = 25) [26].

In addition, the following indicators were used to examine the output and quality of publications in this study: (1) total publications (TP): total number of publications during the observation period; (2) total citations (TC): total citations of publications; (3) TC/TP: average number of citations per publication (CPP).

## Results

### Publication outputs and citation trend

**Fig. 2:**
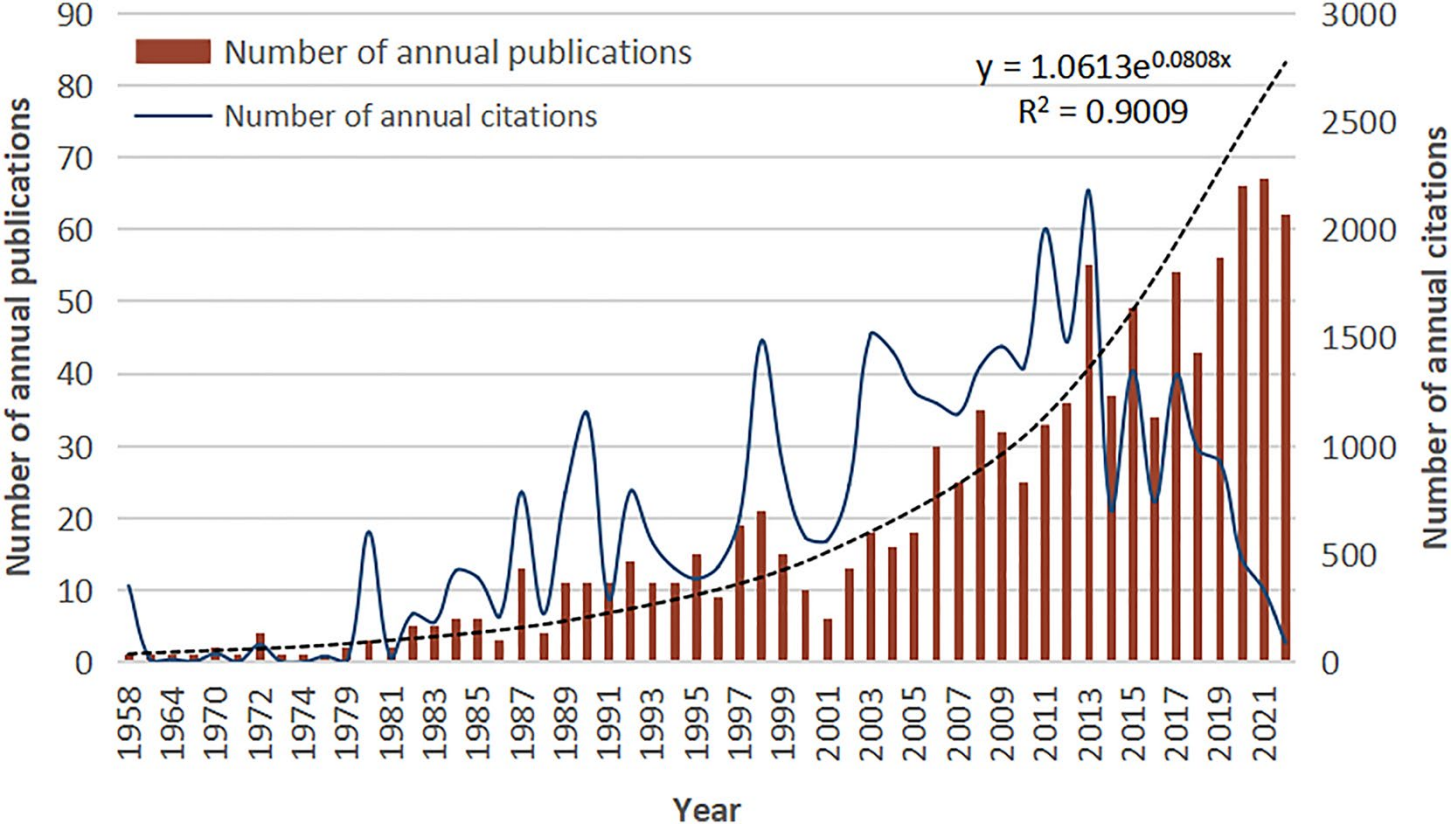
Annual number of the published publications in gastroparesis research

After applying the defined search formula and screening criteria, 1033 publications, including 888 articles (85.96%) and 145 reviews (14.04%), were extracted. Figure 2 shows the annual trends in the number of publications and citations, and the annual growth curve of publications was expressed as a mathematical function (*y* = 1.0613e^0.0808x^). The calculated *R*^2^ of 0.9009 suggested a strong correlation between the number of annual publications and the year of publication [24]. The first article in this field was authored by Kassander and published in 1958 under the title “Gastroparesis” in *Annals of Internal Medicine* [27]. Between 1958 and 1987, articles were published intermittently, with the annual number of articles being less than 10; subsequently, the annual number of publications increased and peaked at 67 in 2021. A similar upward trend was also seen in the annual citations since 1979 and peaked in 2013 (*n* = 2174). These findings indicate that research in the gastroparesis field is gaining attention and will continue to grow in the coming years.

### Contributions of countries

Fifty-three countries or regions participated in gastroparesis-related publications. The USA was the highest contributor, accounting for 645, i.e., 62.44%, of the total publications, which was significantly greater than the contributions of other countries. USA was followed by China (*n* = 86), Australia (*n* = 40), and Belgium (*n* = 38). The country that ranked first in terms of CPP value was Belgium (*n* = 79.47), indicating the high quality of the published research as well as the reference value of the country (Table 1).

**Table 1:** The top 10 most productive countries in gastroparesis research

Rank	Country	TP	Percent (%)	TC	CPP
1	USA	645	62.44	25,890	40.13
2	China	86	8.33	892	10.37
3	Australia	40	3.87	2005	50.13
4	Belgium	38	3.68	3020	79.47
5	Italy	36	3.48	1837	51.03
6	UK	34	3.29	1219	35.85
7	France	28	2.71	1116	39.86
8	Japan	28	2.71	734	26.21
9	Germany	20	1.94	1244	62.20
10	Canada	19	1.84	1029	54.16

*TP* total publications, *TC* total citations, *CPP* number of citations per publication

**Fig. 3:**
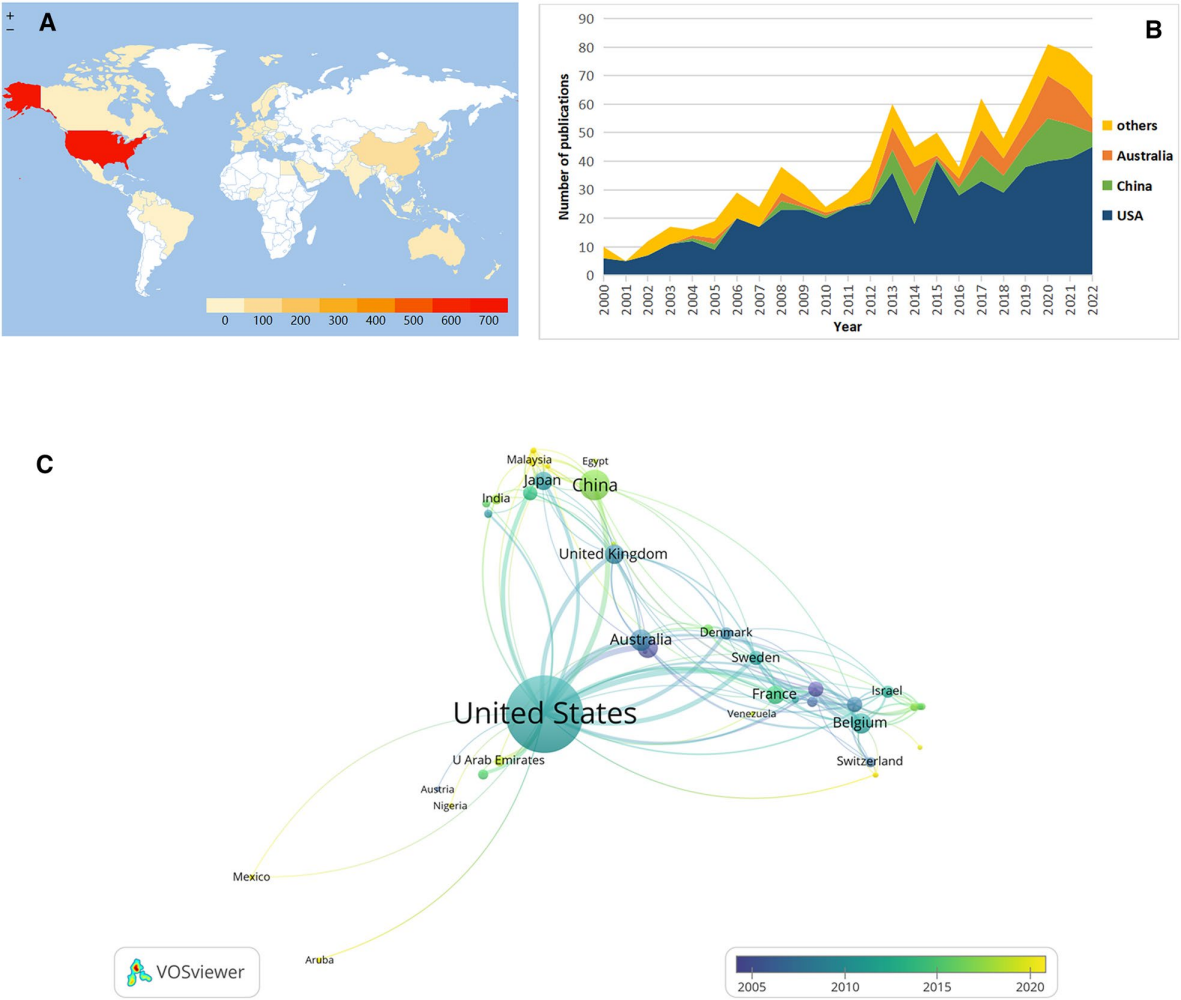
The distribution of countries in gastroparesis research. **A** Distribution of gastroparesis publications in the world map. According to the color gradient in the lower right corner, the color of each country represents the amount of literature published. **B** The distribution trend of the top 3 countries by year. **C** Overlay visualization map of co-authorship among countries

Regional differences in the gastroparesis research field were observed worldwide, with a higher participation from North America, Western Europe, and East Asia (Fig. 3A). The USA was found to be at the center of the cooperation network, maintaining cooperation with 34 countries (Fig. 3C). Although an annual increase was noted in the participation of China and Australia during the recent years, these countries may still have some potential for enhanced cooperation, considering the USA’s long-standing participation and contribution (Fig. 3B).

### Contributions of institutions

**Fig. 4:**
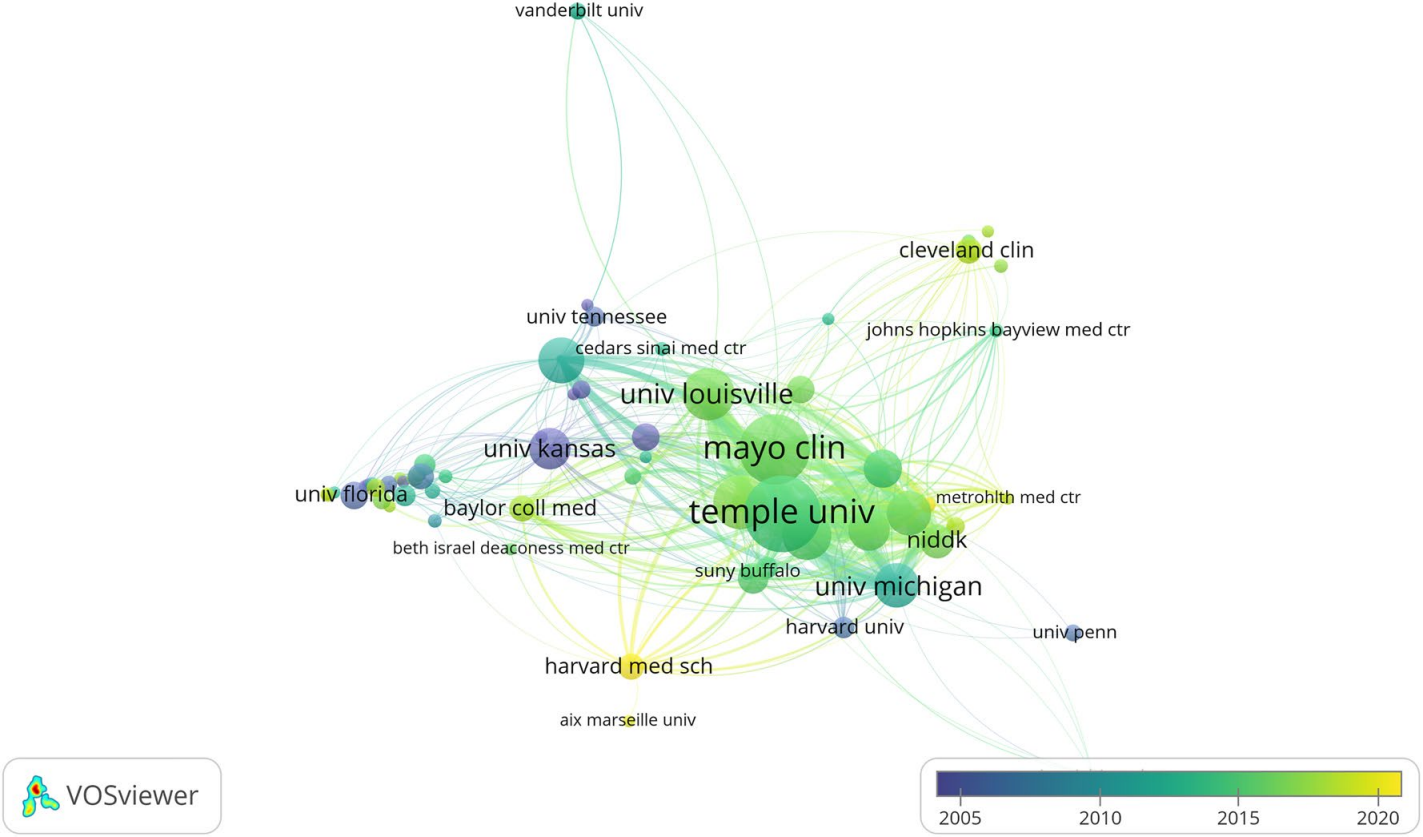
Overlay visualization map of co-authorship among institutions with five or more publications

In all, 966 institutions were involved in publishing in the gastroparesis field, with Temple University contributing 122 publications and accounting for 11.81% of the total publications, a number significantly greater than that of any other institution, followed by Mayo Clinic (*n* = 103), Texas Tech University (*n* = 65), and University of Louisville (*n* = 62) (Table 2). Among them, Mayo Clinic was at the center of the collaborative network, maintaining collaborative relationships with 41 institutions; the closest relationship was with Temple University, with the two institutions collaborating over a total of 52 publications (Fig. 4).

**Table 2:** The top 10 most productive institutions in gastroparesis research

Rank	Institution	Country	TP	TC	CPP
1	Temple University	USA	122	5870	48.11
2	Mayo Clinic	USA	103	5678	55.13
3	Texas Tech University	USA	65	2123	32.66
4	University of Louisville	USA	62	1382	22.29
5	Stanford University	USA	53	2806	52.94
6	University of Mississippi	USA	48	3533	73.60
7	Johns Hopkins University	USA	47	2017	42.91
8	University of Michigan	USA	46	3232	70.26
9	Wake Forest University	USA	40	1932	48.30
10	The University of Kansas	USA	39	3352	85.95

*TP* total publications, *TC* total citations, *CPP* number of citations per publication

### Journal analysis

Research in the field of gastroparesis has been published in 324 journals. Table 3 shows the ten journals with the highest number of articles, covering 397 articles, i.e., 38.43% of the total number of articles. *Neurogastroenterology & Motility* published the most articles (*n* = 89), followed by *Digestive Diseases and Sciences* (*n* = 83), *American Journal of Gastroenterology* (*n* = 40). Among the publishing journals, *Gastroenterology* (IF = 33.88) had the highest IF, while three journals, namely, *American Journal of Gastroenterology*, *Gastrointestinal Endoscopy*, and *Clinical Gastroenterology and Hepatology,* have IF scores above 10. In addition, six journals belonged to the Q1 category, which indicates that research in the gastroparesis field has attracted the attention of many high-quality journals.

**Table 3:** The top 10 Journals with the largest number of publications in gastroparesis research

Rank	Journal	TP	TC	CPP	JCR	IF (2021)
1	Neurogastroenterol Motil	89	2486	27.93	Q2	3.96
2	Dig Dis Sci	83	2953	35.58	Q3	3.49
3	Am J Gastroenterol	40	3518	87.95	Q1	12.04
4	Gastroenterology	36	5393	149.81	Q1	33.88
5	Aliment Pharmacol Ther	34	1791	52.68	Q1	9.52
6	J Clin Gastroenterol	27	783	29.00	Q3	3.17
7	Surgical Endoscopy and Other Interventional Techniques	23	380	16.52	Q1	3.45
8	Gastroenterol Clin North Am	22	652	29.64	Q3	3.87
9	Gastrointest Endosc	22	1082	49.18	Q1	10.40
10	Clinical Gastroenterology and Hepatology	21	986	46.95	Q1	13.58

*TP* total publications, *TC* total citations, *CPP* number of citations per publication, *IF* impact factor, *JCR* journal citation report

### Contributions of authors

**Fig. 5:**
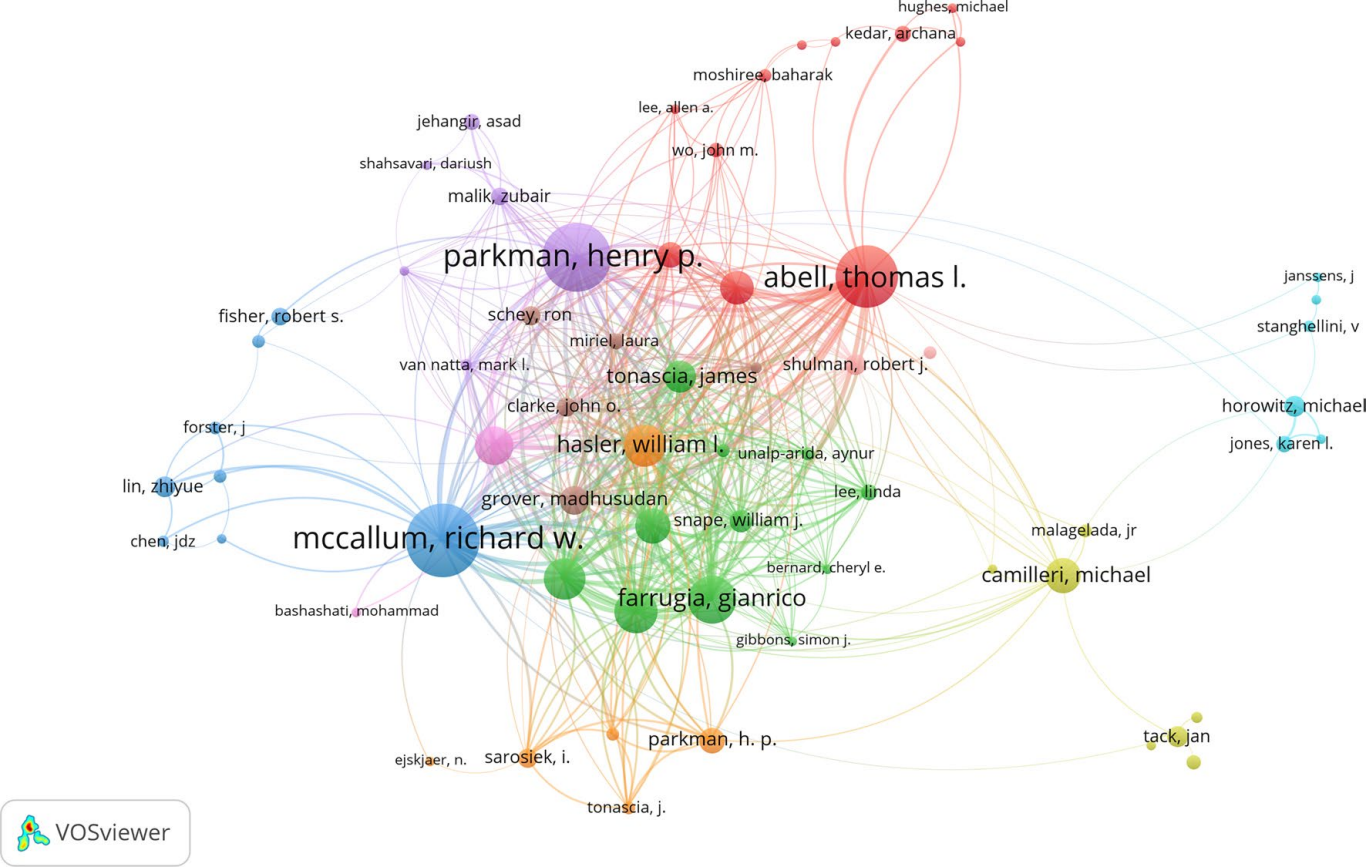
Network visualization map of authors with five or more publications

The data analysis revealed that 3879 researchers have been involved in the research on gastroparesis. Among them, Parkman contributed 96 publications, accounting for 9.29% of the total publications, followed by Abell (*n* = 52), McCallum (*n* = 51), and Farrugia (*n* = 43) (Table 4). In addition, numerous tight-knit research teams have been formed in this field, such as the Farrugia, Pasricha, and Koch team from Temple University, which has contributed to 33 publications pertaining to a wide range of studies involving drug efficacy evaluation [28, 29] and analysis of relevant factors [30, 31] (Fig. 5).

**Table 4:** The top 10 authors and co-cited authors in gastroparesis research

Rank	Author	TP	TC	CPP	Co-cited author	Co-citations
1	Henry P Parkman	96	3676	38.29	Henry P Parkman	826
2	Thomas L Abell	52	2482	47.73	Michael Camilleri	818
3	Richard W McCallum	51	1609	31.55	Thomas L Abell	570
4	Gianrico Farrugia	43	2465	57.33	Richard W McCallum	532
5	Irene Sarosiek	40	777	19.43	Michael Horowitz	490
6	Pankaj J Pasricha	37	1641	44.35	Dennis A Revicki	391
7	William L Hasler	36	1664	46.22	J Tack	378
8	Kenneth L Koch	35	1418	40.51	William L Hasler	299
9	James Tonascia	30	1310	43.67	I soykan	273
10	Richard W Mccallum	29	3206	110.55	Nj Talley	261

*TP* total publications, *TC* total citations, *CPP* number of citations per publication

### Citations

**Fig. 6:**
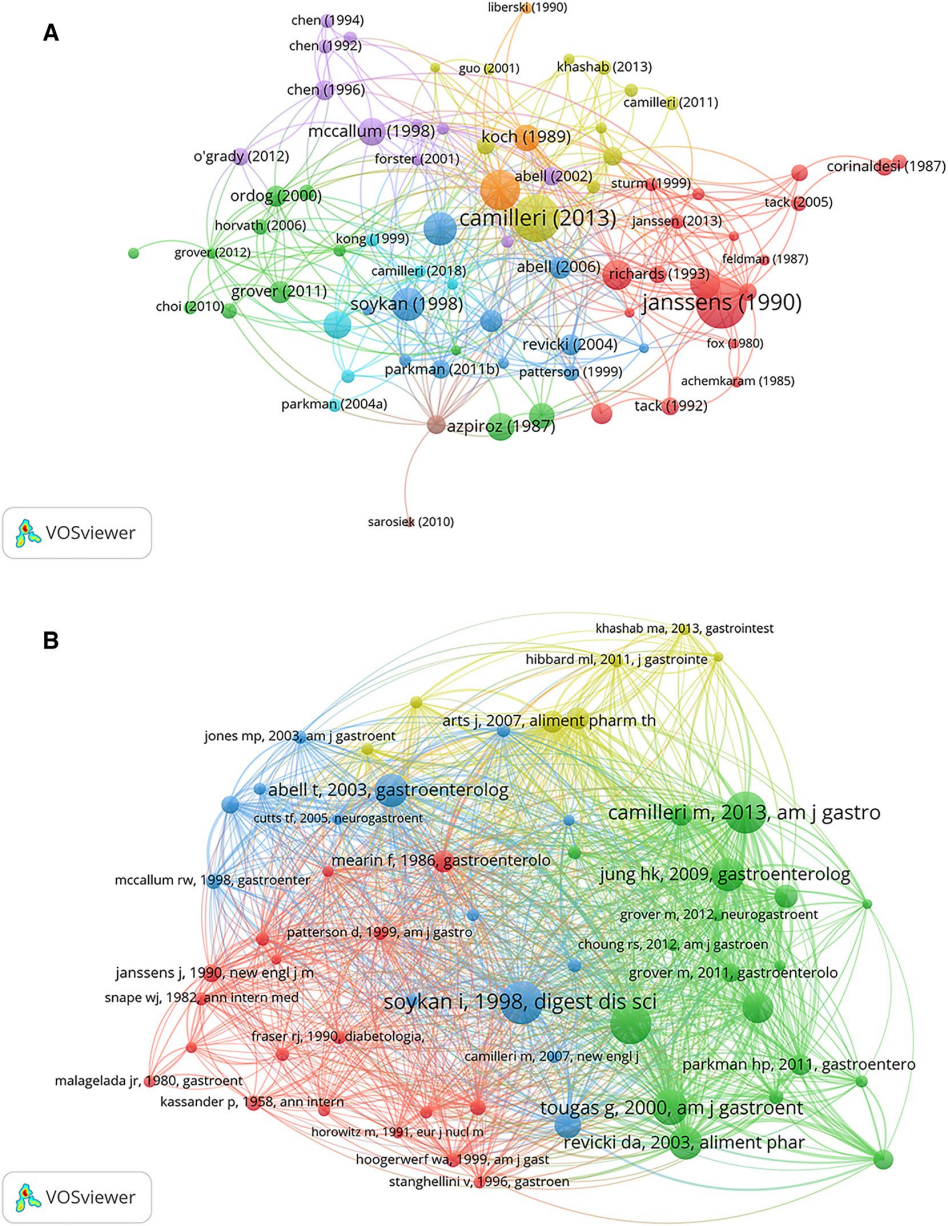
The distribution of citations in gastroparesis research. **A** Network visualization map of citation among documents. **B** Network visualization map of co-citation among documents

Highly cited papers tend to have a significant impact in a particular field [32] and reflect the hot spots and depth of research in the field [33]. In the context of gastroparesis, the article “Improvement of gastric emptying in diabetic gastroparesis by erythromycin: Preliminary studies” published by Janssens in 1990 is the most cited article. This was also the first paper to examine the value of erythromycin in the treatment of DG. The study revealed that erythromycin shortened the gastric emptying time to normal for both liquids and solids, thus providing important evidence for the clinical application and further study of erythromycin [34]. The publications that are ranked second and third in terms of citations are both academic guidelines, with the 2004 article “American Gastroenterological Association technical review on the diagnosis and treatment of gastroparesis” being the first national-level guideline in the field of gastroparesis, offering detailed information on the diagnosis and treatment of the disease. The 2013 article “Clinical guideline: management of gastroparesis” further improved the previous guidelines based on the latest research findings. For example, this article clarifies the three main causes of gastroparesis, and nutritional maintenance and glycemic control have been added to the treatment. In addition, the epidemiological characteristics of the disease [10, 35] and the efficacy of gastric electrical stimulation (GES) [36, 37] are also focus points for researchers in the field of gastroparesis (Table 5, Fig. 6A). In addition, co-citation analysis was performed, and the results are shown in Table 6 and Fig. 6B.

**Table 5:** The top 10 documents in citation analysis of publications in gastroparesis research

Rank	Title	First author	Source	Publication year	TC
1	Improvement of gastric emptying in diabetic gastroparesis by erythromycin. Preliminary studies	J Janssens	N Engl J Med	1990	611
2	Clinical guideline: management of gastroparesis	Michael Camilleri	Am J Gastroenterol	2013	564
3	American Gastroenterological Association technical review on the diagnosis and treatment of gastroparesis	Henry P Parkman	Gastroenterology	2004	466
4	Gastric electrical stimulation for medically refractory gastroparesis	Thomas L Abell	Gastroenterology	2003	381
5	Demography, clinical characteristics, psychological and abuse profiles, treatment, and long-term follow-up of patients with gastroparesis	I Soykan	Dig Dis Sci	1998	381
6	Asymptomatic gastric retention in diabetics (gastroparesis diabeticorum)	P Kassander	Ann Intern Med	1958	336
7	The incidence, prevalence, and outcomes of patients with gastroparesis in Olmsted County, Minnesota, from 1996 to 2006	Hye-Kyung Jung	Gastroenterology	2009	318
8	Gastric pacing improves emptying and symptoms in patients with gastroparesis	Richard W McCallum	Gastroenterology	1998	318
9	Gastric tone measured by an electronic barostat in health and postsurgical gastroparesis	F Azpiroz	Gastroenterology	1987	313
10	Abnormal intestinal motility in diabetics with the gastroparesis syndrome	Michael Camilleri	Eur J Clin Invest	1984	281

*TC* total citations

**Table 6:** The top 10 documents in co-citation analysis of publications in gastroparesis research

Rank	Title	First author	Source	Publication year	TC
1	Demography, clinical characteristics, psychological and abuse profiles, treatment, and long-term follow-up of patients with gastroparesis	I Soykan	Dig Dis Sci	1998	207
2	Clinical guideline: management of gastroparesis	Michael Camilleri	Am J Gastroenterol	2013	201
3	American Gastroenterological Association technical review on the diagnosis and treatment of gastroparesis	Henry P Parkman	Gastroenterology	2004	197
4	Assessment of gastric emptying using a low fat meal: establishment of international control values	G Tougas	Am J Gastroenterol	2000	168
5	The incidence, prevalence, and outcomes of patients with gastroparesis in Olmsted County, Minnesota, from 1996 to 2006	Hye-Kyung Jung	Gastroenterology	2009	159
6	Development and validation of a patient-assessed gastroparesis symptom severity measure: the Gastroparesis Cardinal Symptom Index	Dennis A Revicki	Aliment Pharmacol Ther	2003	159
7	Gastric electrical stimulation for medically refractory gastroparesis	Thomas L Abell	Gastroenterology	2003	158
8	Consensus recommendations for gastric emptying scintigraphy: a joint report of the American Neurogastroenterology and Motility Society and the Society of Nuclear Medicine	Thomas L Abell	Am J Gastroenterol	2008	150
9	Treatment of gastroparesis: a multidisciplinary clinical review	Thomas L Abell	Neurogastroenterol Motil	2006	126
10	Gastroparesis Cardinal Symptom Index (GCSI): development and validation of a patient reported assessment of severity of gastroparesis symptoms	Dennis A Revicki	Qual Life Res	2004	114

*TC* total citations

### Keywords analysis

#### Research hotspots: co-occurrence and clustering analysis of keywords

**Fig. 7:**
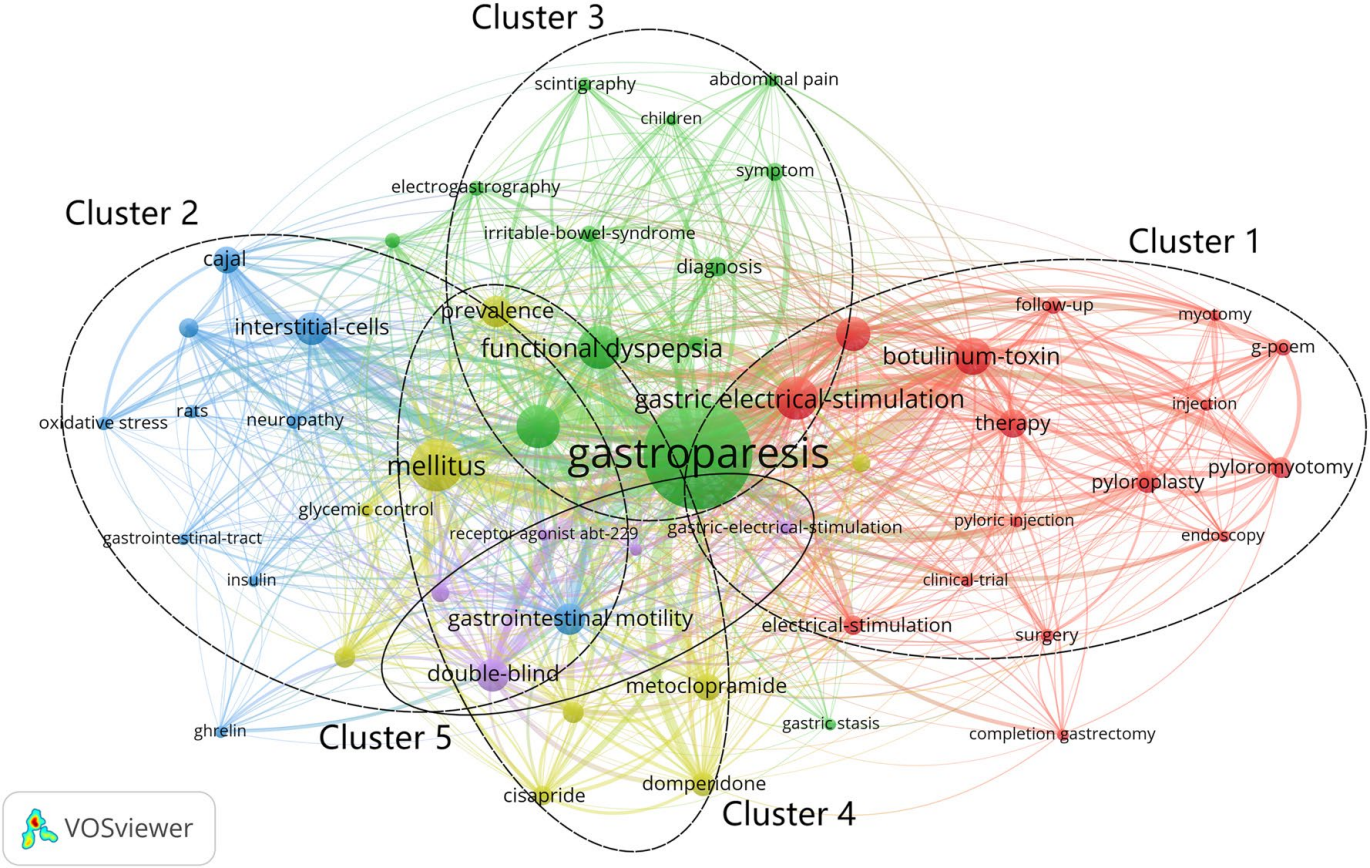
Co-occurrence network visualization map of keyword

High-frequency keywords represent popular topics in a research field [38]. In the current study, 2487 keywords were included. Fifty of these keywords were used at least 15 times. Table 7 shows the top 20 keywords in terms of frequency. Based on the keyword co-occurrence network, the keywords can be divided into five clusters, according to the color (Fig. 7): Cluster 1 (red), diagnostic methods; Cluster 2 (blue), surgical interventions; Cluster 3 (green), pathological mechanism; Cluster 4 (yellow), pharmacological intervention; and Cluster 5 (purple), others, involving “acid breath test,” “botulinum toxin injection,” “double-blind,” etc.

**Table 7:** The top 20 keywords in gastroparesis research

Rank	Keyword	TP	Rank	Keyword	TP
1	Gastroparesis	618	11	Gastrointestinal motility	76
2	Mellitus	181	12	Therapy	64
3	Functional dyspepsia	134	13	Metoclopramide	59
4	Gastric emptying	132	14	Cajal	57
5	Gastric electrical-stimulation	128	15	Domperidone	49
6	Botulinum-toxin	103	16	Pyloroplasty	42
7	Quality of life	86	17	Erythromycin	41
8	Interstitial-cells	84	18	Autonomic neuropathy	38
9	Double-blind	83	19	Pyloromyotomy	38
10	Prevalence	78	20	Cisapride	37

*TP* total publications

#### Research fronts: keyword burst analysis

**Fig. 8:**
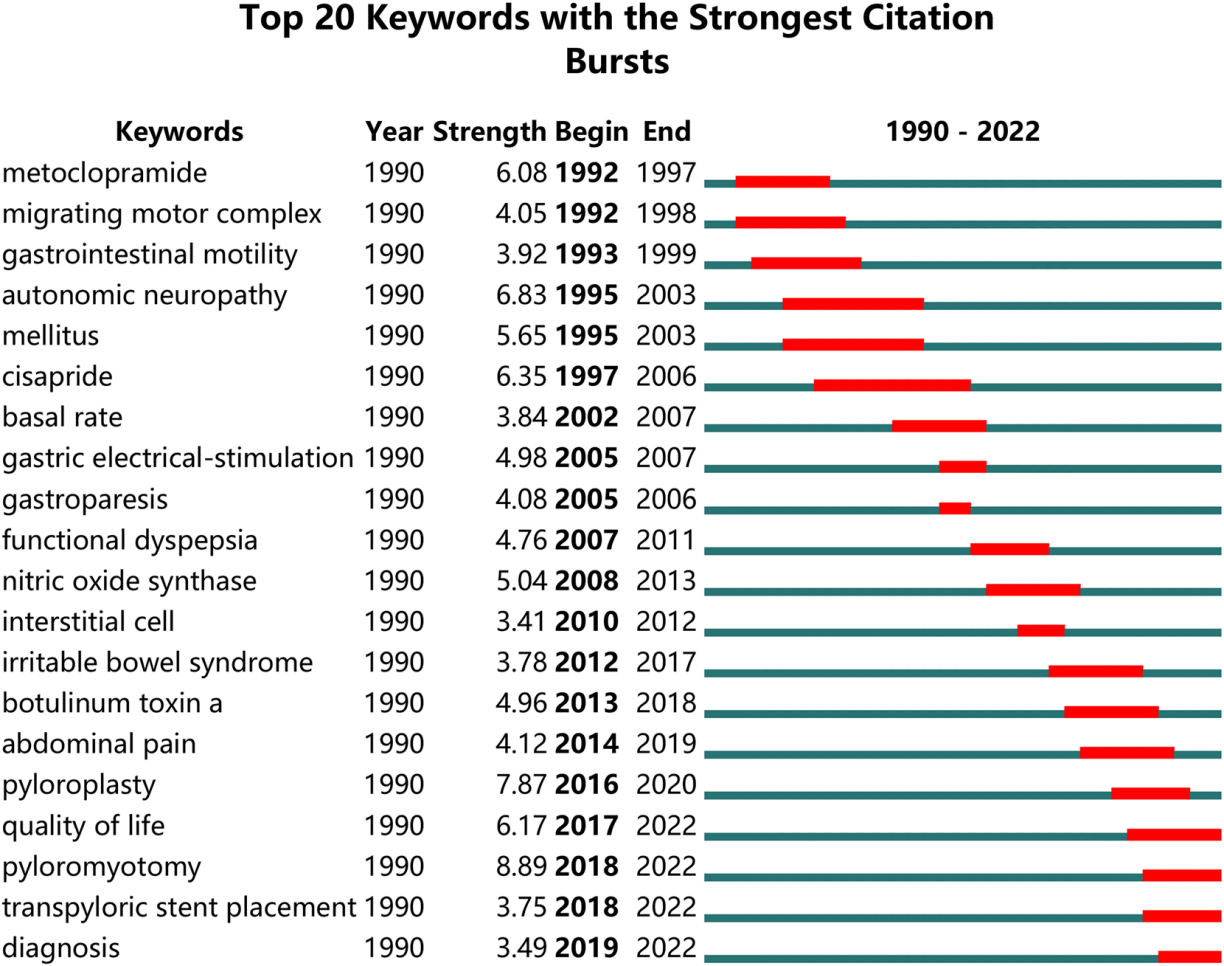
Top 20 keywords with the strongest citation bursts

Keyword burst analysis is used to determine the evolution of research hotspots by analyzing the temporal change characteristics in burst words [39]. Figure 8 shows the top 20 keywords in terms of burst intensity for the period of 1990–2022. The strongest keyword is pyloromyotomy (8.89), and the keyword with the longest burst duration is cisapride (1997–2006). Furthermore, the keywords that continue to explode until 2022 are quality of life, pyloromyotomy, transpyloric stent placement, and diagnosis, which are areas of cutting-edge research in the field of gastroparesis.

## Discussion

In this study, we used the bibliometric method and retrieved 1033 publications related to gastroparesis from the WOSCC database. We analyzed the contributions of countries, institutions, authors, and journals in the gastroparesis field and summarized the research hotspots and future directions. Our search revealed that 53 countries, 966 institutions, 3879 authors, and 324 journals are involved in research in the field of gastroparesis. The United States undoubtedly occupies the leading position, both in terms of the volume of articles published and international collaborations, and it is an important initiator and promoter of gastroparesis-related research. The Temple University, with its research team represented by Farrugia, Pasricha, and Koch, has produced the highest number of articles, making it a regional research center.

A combined analysis of keyword frequency and keyword clustering reveals that the research hotspots mainly revolve around four areas: innovation in diagnostic modalities, change of oral therapeutic agents, choice of surgical interventions, and pathological mechanism. From the analysis of keyword bursts, it can be predicted that future research will mainly be focused on quality of life, diagnostic techniques, pyloromyotomy, and transpyloric stent placement.

### Innovation in the diagnostic approach

The diagnosis of gastroparesis is based on three parameters: (1) symptoms of dyspepsia associated with gastroparesis; (2) absence of abnormalities on gastroscopy; and (3) evidence of the occurrence of DGE [40, 41]. Questionnaires, such as the Gastroparesis Cardinal Symptom Index (GCSI) [42] and the GCSI-Daily Diary (GCSI-DD), have been developed to quantify the severity of symptoms. Gastric emptying scintigraphy (GESc) is currently the diagnostic tool most commonly used for the detection of DGE; in addition, wireless motion capsules (WMC) and gastric emptying breath test (GEBT) have also been clinically validated for their diagnostic value [43].

In GESc, a gamma camera is used to detect the migration of radioisotopes, thereby reflecting the transport characteristics of substances in the gastrointestinal tract [44]. The use of GESc for the investigation of gastroparesis patients was first proposed in 1966 [45]. Because it provides an objective measure of gastric emptying (GE) [46] and offers the advantage of being noninvasive, it has now become the gold standard for the diagnosis of gastroparesis [47]. However, previously, the test lacked standardization due to variations in the food taken and the duration of imaging [48]. This concern was overcome in 2008, when the American Neurogastroenterology and Motility Society and the Society of Nuclear Medicine published the world’s first consensus recommendations on GESc [49], detailing the steps to be taken before and after the examination. In particular, these recommendations provide detailed descriptions of the food composition and ratio of the GE meal, the time and angle of the shot, and the normal values of the parameters’ investigations during the examination. However, low compliance with the GES consensus recommendations is still very common and is a major cause of the underdiagnosis and misdiagnosis of gastroparesis [50]. In addition, some patients may not be able to tolerate solid food [51], and some studies have even shown that liquid GE studies have greater diagnostic value when compared to GE studies with solid food [52]. All these discrepancies have caused considerable problems for the clinical application of GESc.

GESc is not recommended in certain populations, such as children and pregnant women, because of the risk associated with radiation exposure [53]. The procedure also takes a long time per patient, making the test inefficient [54]. Thus, GEBT has become a safe and effective alternative [55]. GEBT is easy to operate, can be performed anywhere, and can be transferred to the field for analysis [56]. In 1993, Ghoos et al. [57] first detected the GE rate by carbon-labeled octanoic acid breath test. Their study creatively proposed three parameters for analysis, namely, GE coefficient, gastric half-emptying time, and stagnation period, and also established GEBT as a reliable and noninvasive test. However, patients with combined diabetes or scleroderma may have false-negative results with GEBT [58]. Another limitation of GEBT is that it does not allow for the identification of the specific area of injury [59]. Apart from GESc and GEBT, WMC is a commonly used technique for assessing gastrointestinal dynamics [44]. The WMC sensor allows for continuous data collection of pH, pressure, and temperature of the gastrointestinal tract for up to 5 days [60]. A study comparing GESc and WMC in gastroparesis assessment suggests that GESc has higher sensitivity, sensitivity, and specificity [61]. However, WMC has a higher diagnostic rate and also identifies abnormalities in extragastric transport [62].

### Change of oral therapeutic agents

With regard to the management of gastroparesis, there is a lack of effective therapeutic medications. Although there are a limited number of clinical studies suggesting superior efficacy of dopamine antagonists over placebo, most of these studies were conducted more than 20 years ago [63]. Thus far, metoclopramide is the only drug approved for marketing in the United States for the treatment of gastroparesis [64]. Metoclopramide is a dopamine receptor antagonist [65] that has antiemetic properties along with prokinetic activity [66]. Its therapeutic efficiency has been confirmed by clinical studies [67, 68]. The dosage form of metoclopramide was upgraded from a tablet to a nasal spray, to prevent vomiting associated with oral intake; this change did not result in any significant decrease in efficacy, although there were gender differences in effects [69]. Nevertheless, the adverse effects of the drug continue to be reported. For example, a study of 34,685 diabetic patients treated with metoclopramide found that the risk of Parkinson’s syndrome increased with the duration of treatment, regardless of whether or not the drug was used for more than 3 months [70]. In fact, in 2009, the USA Food and Drug Administration (FDA) issued a warning that metoclopramide use may lead to irreversible delayed-onset dyskinesia [71]. Dopamine receptor antagonists commonly used in clinical practice also include domperidone [72] and levosulpiride [73]. Another class of drugs used to treat gastroparesis comprises the 5-hydroxytryptamine-4 (5-HT4) receptor agonists [74]. However, non-specific 5-HT4 receptor agonists tend to cause cardiac conduction abnormalities [75], which was a major reason for the withdrawal of cisapride from the US market in 2000 [76]. Prucalopride is highly specific for the 5HT4 receptor, making it safer than earlier 5-HT4 receptor agonists [77]. In a 4-week randomized controlled trial, gastroparesis patients treated with prucalopride showed better GE and significant improvement in symptoms, quality of life, and survival, with only three patients developing adverse effects such as nausea and vomiting [78]. Aprepitant is a neurokinin-1 receptor (NK1R) antagonist previously used for the prevention and treatment of chemotherapy and postoperative nausea [29]. Although current evidence does not show a positive effect of aprepitant on accelerating GE [79], some case reports suggest that aprepitant may be useful in the short term to relieve nausea and vomiting in patients with gastroparesis, while awaiting further evaluation and treatment [80].

### Choice of surgical interventions

Medication is often slow to take effect, with one-third of patients showing no improvement until 1 year after the intervention [81]. The adverse effects of medications also affect the compliance of patients [82]. High-frequency GES is being used as a treatment option for medically refractory gastroparesis [83]. As early as 1963, Bilgutay Am et al. hypothesized that gastrointestinal motility could be induced by electrical stimulation, in analogy to myocardial electrical stimulation for heart block. This was confirmed by experiments in dogs [84]. Since then, animal and clinical trials have been carried out to elucidate the characteristics of the electrical activity of the gastric muscle as well as the optimal frequency and duration of stimulation required [85, 86]. The clinical use of low-frequency pulses is limited by their high power consumption. Therefore, currently, high-frequency pulses are the main form of electrical pulses used in GES [87]. Despite this, the practical application of theory is very difficult. The first GES system, Enterra™ (Medtronics, USA), was not approved by the USA FDA until 2000. To date, this device remains the only one of its kind [88]. A 1-cm-long electrode is surgically placed 10 cm proximal to the pylorus, and the wire is connected to an implanted generator to deliver high-frequency electrical pulses to the stomach, with an amplitude of 5 mA at intervals of 72 ms [89]. A recent meta-analysis suggests that GES treatment significantly improves the frequency of vomiting, gastrointestinal symptoms, and quality of life in patients [90]. A 10-year follow-up found that patients showed significant weight gain after GES, and J-tubes of 89% of the patients could be removed [91]. McCallum et al [92] suggest that GES may be achieved by activating vagal afferent pathways and that it should not be understood simply as “pacing.” GES thus represents a therapeutic option for patients with refractory gastroparesis. However, due to its high cost and invasive nature, the choice of GES for a given patient should be made with great caution [93].

Gastric per oral endoscopic esophageal myotomy (G-POEM) is a recently developed surgical treatment technique. This technique was derived from the successful operation used for the treatment of esophageal achalasia by Inoue et al [94]. Later, in 2013, Mouen A Khashab et al. [95] were the first to report the use of G-POEM for the treatment of gastroparesis. Intraoperative investigation indicates that the length of myotomy was usually shorter than that of cardia achalasia, usually between 1.5 and 2 cm [96]. A meta-analysis of 332 patients treated with G-POEM found a clinical success rate of 75.8% and 85.1%, as determined by GCSI and GES results, respectively [97]. Intraoperative complication rates of G-POEM can reach 5.1%, with postoperative complication rates of 6.8% [98]. The rate of improvement in GCSI appeared to decrease with an increase in the duration of postoperative follow-up, but remained above 50% at 1 year postoperatively [99]. Meanwhile, the mean duration of surgery and hospitalization was significantly lower in G-POEM compared with pyloromyotomy/pyloroplasty. [100]

A neurotoxic protein produced by *Clostridium botulinum* [101], botulinum toxin, affects cholinergic nerve contractility at low doses and inhibits acetylcholine release. Intrapyloric botulinum toxin A injection (IPBTI) is administered to resolve the imbalance between acetylcholine and nitric oxide [102] levels, thereby relieving pyloric spasm in patients with gastroparesis [103]; this is because pyloric spasm is thought to be a possible contributing factor to the development of DG [104]. However, although several reports suggest the efficacy of IPBTI [105, 106], a systematic review of pertinent reports yielded contradictory results, highlighting the low quality of studies currently conducted around IPBTI [107].

### The epidemiology of gastroparesis

In addition to the above research hotspots, we found that epidemiological terms such as quality of life and prevalence also appeared as high-frequency keywords. The Rome Foundation Global Epidemiology Study (RFGES) is a large clinical study involving 33 countries and 73,076 subjects, with research findings published in *Gastroenterology* in 2021. It assessed the prevalence and burden of functional gastrointestinal disorders by providing the Rome IV Diagnostic Questionnaire and an 80-item supplemental questionnaire [108]. Huang et al. used the RFGES database to further analyze and obtain epidemiological evidence on the gastroparesis-like symptoms (GPLS) population. The global prevalence of GPLS was found to be 0.9% in general, with higher prevalence in the United States, Italy, Brazil, Russia, Canada, South Korea, and China than in other countries, which is consistent with the results of the country contribution of our study. In addition, GPLS patients often present an overlap of symptoms with epigastric pain syndrome or irritable bowel syndrome, which explains why functional dyspepsia ranked the third in the frequency of keywords [2].

There are some limitations to this study. First, the literature search is limited to the WOSCC database, and although WOSCC can cover the majority of studies in the field of gastroparesis, there may still be individual qualified literature excluded. Second, as the database is dynamically updated, our study should be updated at the same time. Third, non-English publications are excluded, which may lead to retrieval incompleteness.

## Conclusion

The number of publications on gastroparesis has steadily increased over the last four decades. The USA, Temple University, and Parkman were the most productive country, institution, and author respectively. *Neurogastroenterology & Motility* published the most articles. These publications have mainly covered four major domains, namely, innovation in diagnostic modalities, change of oral therapeutic agents, choice of surgical interventions, and pathological mechanisms. Future research on gastroparesis should focus on the quality of life of patients, diagnostic techniques, pyloromyotomy, and transpyloric stent placement.

## Data Availability

Data is available upon reasonable request.

## Abbreviations

5-HT4: 5-Hydroxytryptamine-4
CPP: Number of citations per publication
DG: Diabetic gastroparesis
DGE: Delayed gastric emptying
FDA: Food and drug administration
GCSI: Gastroparesis cardinal symptom index
GCSI-DD: GCSI-daily diary
GE: Gastric emptying
GEBT: Gastric emptying breath test
GES: Gastric electrical stimulation
GESc: Gastric emptying scintigraphy
GPLS: Gastroparesis-like symptoms
G-POEM: Gastric per oral endoscopic esophageal myotomy
IF: Impact factor
IG: Idiopathic gastroparesis
IPBTI: Intrapyloric botulinum toxin a injection
JCR: Journal citation report
MeSH: Medical subject headings
NK1R: Neurokinin-1 receptor
PG: Postoperative gastroparesis
RFGES: Rome Foundation Global Epidemiology Study
SCIE: Science citation index-expanded
TC: Total citations
UK: United Kingdom
USA: United States of America
TP: Total publications
WMC: Wireless motion capsules
WOS: Web of science
WOSCC: The WOS Core Collection
